# MicroRNA as Potential Biomarkers and Their Pathogenesis in Multiple System Atrophy

**DOI:** 10.3390/ijms27041878

**Published:** 2026-02-15

**Authors:** Ming-Che Kuo, Shao-Ying Cheng, Meng-Ling Chen, Ruey-Meei Wu

**Affiliations:** 1 Department of Medicine, National Taiwan University Cancer Center, Taipei 106037, Taiwan; a00568@ntucc.gov.tw; 2 Institute of Clinical Medicine, College of Medicine, National Taiwan University, Taipei 100233, Taiwan; 3 Department of Neurology, National Taiwan University Hospital Bei-Hu Branch, Taipei 108206, Taiwan; 4 Department of Neurology, National Taiwan University Hospital, Taipei 100225, Taiwan; dennette99@gmail.com; 5 Center of Parkinson and Movement Disorder, National Taiwan University Hospital, Taipei 100227, Taiwan; 6 Department of Neurology College of Medicine, National Taiwan University, Taipei 100233, Taiwan

**Keywords:** multiple system atrophy, microRNA, pathoetiology, differential diagnosis, biomarker, biofluid

## Abstract

Multiple system atrophy (MSA) is a rare, rapidly progressive neurodegenerative disorder characterized by autonomic dysfunction, Parkinsonism, and cerebellar ataxia. While the pathological hallmark of MSA is the accumulation of α-synuclein in oligodendrocytes and formation of glial cytoplasmic inclusions (GCIs), the precise etiopathogenesis, accurate biomarkers, and promising therapeutic targets remain elusive. This review synthesizes current evidence regarding the role of microRNAs (miRNAs) in MSA, focusing on how small non-coding RNAs mediate gene–environment interactions contributing to disease pathogenesis. We explore dysregulated miRNA profiles in MSA, their impact on α-synuclein aggregation, neuroinflammation, demyelinating process, and oligodendrocyte dysfunction, and their potential as biomarkers and therapeutic targets. Understanding the complex interplay between miRNAs, genetic susceptibility, and environmental factors may provide critical insights into MSA pathophysiology and open new avenues for therapeutic intervention.

## 1. Introduction

Multiple system atrophy (MSA) is a sporadic, adult-onset neurodegenerative disorder with an estimated incidence of 0.6–0.7 per 100,000 person-years [1,2]. Clinically, MSA is categorized into two subtypes: MSA with predominant Parkinsonism (MSA-P) and MSA with predominant cerebellar ataxia (MSA-C) [3]. The disease is characterized by rapid progression with a median survival of 6–10 years from symptom onset [4].

The neuropathological hallmark of MSA is the presence of glial cytoplasmic inclusions (GCIs) containing misfolded α-synuclein in oligodendrocytes [5,6]. Despite significant advances in understanding MSA pathology, the precise mechanisms underlying disease initiation and progression remain incompletely understood. While MSA is generally considered a sporadic disorder, emerging evidence suggests that both genetic and environmental factors contribute to disease susceptibility and progression [7,8,9].

This review focuses on the role of a type of small non-coding RNA (sncRNA) called microRNA (miRNA) in the pathophysiology and etiology of MSA. We summarize current knowledge regarding miRNA and its biogenesis, updated datasets of dysregulated biofluid or brain-derived miRNA profiles in MSA, their impact on key pathological processes, and their potential as biomarkers and therapeutic targets. These points can provide additional information on the current research on miRNA in the field of MSA.

## 2. MicroRNA Biogenesis and Function

### 2.1. MicroRNA Biogenesis

MicroRNAs (miRNAs) are small, non-coding RNA molecules approximately 22 nucleotides (19–24 in) in length that regulate gene expression post-transcriptionally by binding to complementary sequences in the 3′ untranslated regions (UTRs) of target mRNAs. This leads to mRNA degradation or translational repression [10]. miRNAs were first discovered in the nematode Caenorhabditis elegans by Victor Ambros and Gary Ruvkun, who were awarded the 2024 Nobel Prize in Physiology or Medicine for this discovery [11]. In recent years, miRNAs have emerged as critical regulators of neurodevelopment and neurodegeneration [12]. Importantly, miRNAs function at the interface of genetic and environmental factors, potentially mediating gene–environment interactions relevant to neurodegenerative diseases [13].

MicroRNAs are transcribed from genomic DNA as primary miRNAs (pri-miRNAs) by RNA polymerase II or III [14]. Pri-miRNAs are processed in the nucleus by the Drosha-DGCR8 complex into precursor miRNAs (pre-miRNAs), which are approximately 70 nucleotides in length and form a hairpin structure [15]. Pre-miRNAs are then exported to the cytoplasm by Exportin-5 [16] and further processed by the endonuclease Dicer into mature miRNA duplexes [17]. One strand of the miRNA duplex is incorporated into the RNA-induced silencing complex (RISC), which guides the complex to target mRNAs [18].

### 2.2. MicroRNA Function

Mature miRNAs primarily function by binding to complementary sequences in the 3′ untranslated regions (UTRs) of target mRNAs, leading to translational repression or mRNA degradation [19,20]. However, accumulating evidence indicates that miRNAs can also activate gene expression under certain conditions. For example, Vasudevan et al. demonstrated that miR-369-3 can upregulate translation of target mRNAs during cell cycle arrest [21]. Additionally, some studies have reported that miRNAs may activate gene expression by interacting with promoter regions [22].

The specificity of miRNA targeting is largely determined by the seed region, a sequence of 2–8 nucleotides at the 5′ end of the miRNA [23]. A single miRNA can regulate hundreds of target genes, and conversely, a single mRNA can be regulated by multiple miRNAs, creating complex regulatory networks [24]. The mature application of microRNAs in medical and pharmaceutical fields is under extensive exploration. However, a single mature miRNA can be generated from multiple distinct gene loci in the genome as a result of gene duplication events [25]. Groups of miRNAs with highly similar sequences—often sharing a conserved seed region—are classified as miRNA families [26]. These families typically arise from common ancestral genes and may include members located at different genomic loci. It imposed a technical barrier for us to generate miRNA knock-out (KO) systems as disease models.

### 2.3. MicroRNAs in the Central Nervous System

Surprisingly, microRNAs are abundantly expressed in the central nervous system (CNS) and play critical roles in neurodevelopment, neuronal function, and neuroprotection [27]. Distinct miRNA expression patterns have been observed in different brain regions and cell types, reflecting their specialized functions [28]. In the adult brain, miRNAs regulate synaptic plasticity, neurotransmission, and neuronal survival [29]. Dysregulation of miRNA expression and function has been implicated in various neurodegenerative disorders, including Alzheimer’s disease (AD), Parkinson’s disease (PD), and amyotrophic lateral sclerosis (ALS) [30]. The differentiation of various CNS cell lineages—including neurons, astrocytes, and oligodendrocytes—is also regulated by the coordinated action of multiple miRNAs [31]. Identifying cell-type-specific or enriched miRNAs, many of which remain unknown, will facilitate the discovery of CNS disorder-associated miRNAs in cerebrospinal fluid (CSF) and peripheral blood. These miRNAs hold promise as diagnostic biomarkers and as mechanism-based therapeutic targets, including MSA.

## 3. Dysregulated MicroRNA Profiles in MSA

### 3.1. Circulating MicroRNAs as Potential Biomarkers for MSA

Circulating microRNAs (miRNAs) in biofluids such as blood and cerebrospinal fluid (CSF) have emerged as promising non-invasive biomarkers for neurodegenerative disorders [32]. Various types of sncRNAs, including PIWI-associated RNA (piRNA), circular RNA (cirRNA), and tRNA fragments (tRF), have been reviewed in PD [33]. However, miRNA studies in MSA warrant more attention since both diseases share common pathological proteins but exhibit distinct cell-specific pathology and different clinical manifestations.

#### 3.1.1. Blood-Based Fluid MicroRNA Biomarkers of MSA

To date, four studies have assessed blood-derived miRNA and have profiled MSA using slightly different study designs. Arrays with an increasing number of miRNA targets have been utilized together (Table 1) [34,35,36,37].

Vallenlunga et al. used Taqman Low-Density Array (TLDA) technology to discover differentially expressed serum-derived miRNAs from a discovery set of nine cases of MSA (six with MSA-P and three with MSA-C), six cases of PD, and five cases of HC. The results were validated by rt-qPCR in a growing sample size of 34 cases of MSA, 31 cases of PD, and 30 cases of HC [34]. The differentially expressed miRNAs between MSA and PD groups had higher levels of miR-24, miR-34b, and miR-148b in MSA. When combining MSA and PD as a united group of alpha-synculeinopathy, higher levels of miR24, miR-223*, and miR-324-3p and lower levels of miR-339-5p were found compared to HC, suggesting shared miRNA traits between the two diseases. Inter-group comparisons further found a decreasing trend of miR-24 in MSA, PD, and HC, and miR-148b in MSA, HC, and PD in order. These miRNA candidates largely participated in fundamental biological processes, including cell cycles and apoptosis.

Kume et al. used microarrays to investigate serum-derived miRNAs in 10 cases of MSA versus six cases of HC and identified 50 upregulated and 17 downregulated miRNA candidates [35]. However, only two miRNAs (miR-16 and miR-223) went through the rt-qPCR validation, and no significant differences were yielded between groups. The author claimed that similar findings of upregulated miR-223 are compatible with the study led by Vallenlunga et al., identifying that miR-223* was upregulated in MSA and the combined MSA and PD group versus HC. Here, miR-223 and miR-223* are two different strands derived from the same precursor miRNA molecule. miR-223, referred to as the “mature” or “guide” strand, is the dominant strand. In contrast, miR-223*, known as the “passenger” strand, is less abundant and has its own distinct set of target genes and functions.

Uwatoko et al. used microarrays to analyze plasma-derived miRNAs to distinguish between patients with MSA and PD [36]. They chose 11 miRNAs (4 upregulated and the top 7 downregulated miRNAs) to perform rt-qPCR. After excluding five miRNAs due to the primer dimer formation and suboptimal amplification process, they validated three miRNAs that could differentiate MSA subtypes from PD and healthy controls with complex combinations. Notably, miR-671-5p was the only miRNA able to distinguish MSA-C from MSA-P; in fact, lower levels were expressed in MSA-P and PD than those in MSA-C and HC. Additionally, miR-19b-3p was found at higher levels in PD than in both MSA subtypes and HC, while miR-24-3p was higher in PD than in MSA-C but did not change significantly compared to MSA-P and HC. These findings highlight the heterogeneity of blood-derived miRNAs. Furthermore, the study observed a correlation between the levels of hsa-miR-19b-3p and hsa-miR-24-3p, suggesting a commonly involved regulatory network among circulating miRNAs. This complexity may arise from miRNAs secreted by organs outside the CNS, such as the peripheral nervous system or other peripheral tissues, which can influence CNS functions directly or indirectly [38].

Pérez-Soriano et al. used a more extensive microarray panel to analyze serum-derived differentially expressed miRNAs between MSA, PD, and HC [37]. In a discovery set of 20 cases of MSA and 40 cases of HC, they identified the top 25 dysregulated miRNAs, including 19 upregulated and 6 downregulated miRNAs. They strategically choose to validate 9 out of 25 miRNAs, including miR-106a-5p, miR-122-5p, miR-16-5p, miR-24-3p, miR-7641, miR-191-5p, hsa-let-7b-5p, miR-93-5p, and miR-17-5p, all of which were upregulated by rt-qPCR in the same cohort compared to 19 patients with PD. This was also performed in an additional cohort with 20 cases of MSA, 18 cases of PD, and 20 cases of HC. In the end, only upregulated miR-191 and miR-7641 were able to differentiate MSA from PD. Moreover, the DIANA MirPath KEGG pathway showed that the most relevant miRNAs are greatly involved in fatty acid metabolism, reflecting the pathological involvement of myelin-forming oligodendrocytes consuming fatty acids in the CNS. Using the REACTOME pathway by miRNet, the network analysis showed that the majority of pathways fall into the categories of NOTCH3 translation, processing, and signaling.

Although the array panel can support and increasingly measure a considerable number of miRNA targets, very few miRNAs have been identified as universally present across blood-based miRNA studies on MSA. In fact, only miR-24 or miR-24-3p has been commonly identified. This reflects the dilemma of miRNA studies in this field: microarrays can generate false-positive results, and it is inevitable that many miRNA candidates identified using array-based technology are lost during validation work. Furthermore, double confirmation cannot be performed on the RT-qPCR platform.

#### 3.1.2. CSF-Based Fluid MicroRNA Biomarkers of MSA

Two studies focus on CSF-derived miRNA biomarker research. Marques et al. investigated 10 selected miRNA expressions (miR-19a, miR-19b, miR-24, miR-30c, miR-34b, miR-34c, miR-132, miR-133b, miR-148b, and miR-205) in CSF samples by rt-qPCR from MSA patients, primarily focusing on known miRNAs targeting PD-related genes, such as SNCA, LRRK2, VPS35, ATP13A2, PARK2, or PARK7 [39]. They identified four dysregulated miRNAs out of ten candidates, including miR-19a-3p, miR-19b-3p, miR-24-3p, and miR-34c-5p. Of these, only miR-24-3p was downregulated in both PD and MSA groups compared to HC, with non-significant, lower levels observed in the MSA group. Moreover, miR-24-3p levels also correlated with ataxic symptoms in MSA, suggesting its potential as a clinically relevant diagnostic biomarker. The other three miRNAs were also expressed at lower levels in MSA compared to PD. Additionally, miR-205 was upregulated in PD compared to HC, contrasting with the downregulation of miR-24-3p in PD. Although the combined diagnostic power of these differentially expressed miRNAs was not assessed, their distinct expression profiles suggest potential for inter-group differentiation.

Starhof et al. also identified a potential CSF biomarker panel among a discovery set of 10 cases of MSA, 10 cases of PD, 10 cases of PSP, and 10 cases of HC, and a validation set of 29 cases of MSA, 37 cases of PD, 32 cases of PSP, and 23 cases of HC [40]. A total of 46 miRNAs, consisting of 26 differentially expressed miRNAs and an additional 20 miRNAs with potential importance, were examined in the validation set. Spike-in controls of cel-miR-54-3p and cel-miR-238-3p were chosen as normalizers, and no internal controls were managed. After multiple corrections and adjustments of clinical parameters, such as gender and age, eight miRNAs, including five downregulated (miR-106b-5p, miR-184, miR-331-5p, let-7b-5p, and miR-99a-5p) and three upregulated (miR-218-5p, miR-34c-3p, and miR-7-5p) miRNAs, were differently expressed among cases of PD and atypical Parkinsonism, including MSA and PSP. Finally, two differentially expressed miRNAs (miR-9-3p and miR-106b-5p) differentiated PD from MSA. Interestingly, miR-7-5p was the most representative miRNA in two distinct sets: it differentiated between PD and HC (miR-7-5p, miR-331-5p, and miR-145-5p), and another distinguishing MSA was found in HC (miR-7-5p, miR-34c-3p, and let-7b-5p). This suggests that miR-7-5p has a role in alpha-synucleinopathy rather than disease-specific pathoetiology. The best distinguishers of MSA and PD are miR-9-3p and miR-106b-5p, which putatively target Protein Processing in the Endoplasmic Reticulum pathway and the TGF-beta signaling pathway. Of note, while miR-9-3p and miR-145-5p did not pass the multiple comparison test individually in the statistical analysis, they still had critical weight in distinguishing between diseases. Moreover, this study also examined plasma-derived miRNAs using the same technology, but the discriminating power and the similarity between plasma and CSF disease-representative miRNAs from the same individual are less prominent.

This finding suggests that certain miRNAs may be involved in shared or overlapping pathophysiological pathways in both diseases, while also participating in disease-specific processes.

#### 3.1.3. Perspectives of Biofluid MicroRNA Biomarkers of MSA

Studies on circulating cell-free miRNAs in blood and CSF have commonly identified members of the miR-19, miR-24, and miR-34 families as potential biomarkers of differential diagnosis for MSA and related disorders. KEGG pathway analysis indicates that these miRNAs regulate genes involved in the PI3K-Akt signaling pathway, which is crucial for cell survival and growth. Specifically, miR-19a/19b are implicated in immune responses via the NF-κB pathway and target genes such as PTEN, while miR-24-3p is involved in oxidative stress response and DNA damage repair through p53-related pathways. MiR-34c-5p, a classical tumor suppressor of miRNA, is enriched in pathways related to cellular senescence, Wnt signaling, and Notch signaling.

The aforementioned factors present significant challenges in the use of circulating blood-derived miRNAs as reliable biomarkers for MSA. To explore novel, disease-relevant microRNA candidates, the use of high-throughput, non-hypothetical approaches is required. Examples include next-generation sequencing [41]—based RNA sequencing, which is among the approaches that are currently lacking. Several dilemmas need to be solved. Since environmental exposure and cellular stress can induce epigenetic modifications affecting miRNA expression, the interpretation of circulating miRNA studies is challenging due to external and intrinsic confounders that are difficult to control during participant enrollment [42]. Currently, no disease-specific miRNAs have been addressed and recognized in any neurodegenerative diseases, including AD, PD, or MSA. Several critical issues remain: inter-platform consistency is lacking, proper normalization methods are needed, and there is a lack of stable miRNA controls.

First, the inter-platform reproducibility between different arrays is low. The correlation coefficient between different arrays is generally lower than 0.5, as shown in a previous study [43]. Even the median coefficient correlation between microarray and rt-qPCR is 0.7 at best [44]. The correlation coefficient between rt-qPCR and NGS, or between the microarray and NGS, is also questionable. It is not surprising that the surviving miRNA candidates after rt-qPCR validation barely remain significant, and the shared candidates of up- or down-regulated miRNAs across studies are extremely limited.

Second, the lack of an optimal consensus on microRNA detection, quantification, and normalization method is catastrophic for data integration and comparison [43]. In array-based methods, microarray performance may be compromised based on the design of probes during hybridization [45]. In the RT-qPCR-based approach, there are three primer-based methods using stem-loop-shaped primers (Applied Biosystems): locked nucleic acid primers (Exiqon), and poly-A-tailed primers (QIAGEN, Stratagene). The accuracy of capturing a single miRNA copy among total RNA within the sample largely depends on the specificity of the primer design.

There are also two other types of quantification methods: absolute and relative quantification. Absolute quantification is determined by the input copy number and compares the PCR signal to the standard curve. The more commonly used relative quantification compares the PCR-derived cycle threshold (Ct) value of target miRNAs with supposedly stably expressed endogenous miRNA controls from the same specimen [46]. However, there is no universally accepted endogenous, intrinsic, or internal control. Biofluid miRNA studies summarized in Table 1 used completely different control miRNAs for normalization. A few studies did not even address the miRNA normalizer they applied in the rt-qPCR experiments [47]. This major discrepancy indicates the fundamental difficulty of conducting external validation and meta-analysis in the future.

In addition, the microRNA targets in previous studies are primarily chosen from hypothesis-based approaches, which are either PD-related or *SNCA*-related candidates. Also, previous studies only detected limited, known microRNA targets through relatively low-throughput array-based or rt-qPCR-based platforms. Additionally, the variation in internal and external reference miRNAs and normalization approaches across different studies can impact reproducibility, even when differentially expressed miRNAs are validated by RT-qPCR [48].

Finally, the major problem of biofluid-based miRNA studies is the absence of high-standard diagnosis based on pathology-proven cases of MSA. This can largely diminish the opportunity for applicable clinical utility in the future. However, there are still a few miRNA studies based on the brain mentioned in the following section.

Although MSA and PD are both α-synucleinopathies with a high overlap in early clinical features, only a limited subset of miRNAs currently demonstrates true discriminatory potential between these diseases. Among these, circulating miRNAs detected in serum or plasma, including miR 24, miR 34b, miR 148b, miR 7641, and miR 191 5p, together with CSF-based miRNA panels, represent the most promising candidates for differential diagnosis [34,36,37,40]. Notably, the CSF miRNA combination miR 9 3p and miR 106b 5p demonstrated moderate discriminatory power for distinguishing MSA from PD, with an AUC of 0.73, a 95 percent confidence interval ranging from 0.572 to 0.858, and a p value of 0.00878 [40]. Nevertheless, systematic reporting of diagnostic performance metrics, including AUC, sensitivity, and specificity, remains insufficient, particularly for blood-based markers. Future studies should, therefore, prioritize well-powered validation cohorts, standardized normalization strategies, and combinatorial miRNA panels to improve disease specificity and facilitate clinical translation.

### 3.2. MicroRNA Expression in MSA Brain Tissues

To date, four studies have investigated microRNA (miRNA) expression profiles in post-mortem brain tissue from MSA patients, with a link anticipated between CNS-derived miRNAs and accessible circulating miRNA biomarkers. Other studies have been inhibited by cost and prevalence of biomarkers, which are greatly hindered and discouraged by the invasiveness of investigations.

Ubhi et al. performed miRNA profiling via 543 miRNAs in the frontal cortex from 3 patients with MSA and atypical Parkinsonian syndrome compared to 4 cases of HC. Forty-six significantly dysregulated miRNAs of MSA were identified [47]. miRNA profiles were also compared among five different types of MSA transgenic (tg) mouse models versus non-transgenic mice using a PCR array involving 88 miRNAs. Among the final list of 47 miRNAs, miR-96 was significantly upregulated in both MSA brains and one mouse model of MBP1-hαsyn-tg mice via rt-qPCR validation. Functional studies of putative target genes of miR-96 were also performed, as discussed in a later section. However, no reasonable explanation is given to answer the question of how miR-96 was the only chosen miRNA, and why other significantly upregulated miRNAs were omitted among the final list of 47 miRNAs. This included familiar miRNAs reported in the biofluid section, such as miR-19b, miR-19b, and miR-24.

The frontal cortex might be late involved during the disease course of MSA. Lee at al. examined cerebellum-derived miRNA via microarray and identified 9 downregulated (miR-129-3p, miR-129-5p, miR-132, miR-206, miR-337-3p, miR-380, miR-410, and miR-409-5p, miR-433) and 2 upregulated (miR-199a-5p, miR-202) miRNAs [49]. However, they chose only one upregulated miRNA, miR-202, to validate its expression level in brain tissues using rt-qPCR. Notably, they used Western blot to conclude that *Oct1*, one of the six putatively targeted gene loci among four genes of miR-202, is authentically modulated by miR-202. Since the authors used cerebellar specimens, we would expect future functional studies using cerebellar-specific cell models, such as Purkinje cells or oligodendrocyte lineage cells, to elucidate the etiological relevance of miR-202 in MSA.

Wakabayashi et al. conducted a comprehensive analysis of miRNA expression in multiple formalin-fixed paraffin-embedded samples from brain regions affected in MSA, including the striatum, cerebellum, and substantia nigra—regions more directly implicated in MSA pathology than the frontal cortex [50]. They identified region-specific miRNA alterations, with the most pronounced changes observed in the striatum. Notably, the miR-129 family (including miR-129-3p, miR-129-5p, and miR-129-2-3p), which targets genes involved in oligodendrocyte differentiation and myelination, was significantly downregulated in the striatum of MSA patients. Functional pathway analysis using KEGG and Gene Ontology (GO) enrichment revealed that these dysregulated miRNAs, including miR-129 and miR-183 families, are mainly involved in cell-cycle-related pathways (i.e., apoptosis, cell cycle regulation, and cell proliferation), immune-related pathways (i.e., Toll-like receptor signaling pathway), and neuronal development in sensory organs (i.e., eye and olfactory epithelium). Notably, the downregulation of miR-129-3p and miR-129-5p in the cerebellum was confirmed in another study [49]. The downregulation of these two miR-129 family miRNAs was also found in the pons, indicating their cross-regional role in MSA brains.

**Table 1:** Dysregulated biofluid microRNA profiles in MSA.

First Author and Publication Year	Sample Resources	Sample Size	MicroRNA Detection	Differentially Expressed miRNAs
Vallenlunga (2014), Italy [34]	Serum	Discovery: 9 MSA (6 MSA-P and 3 MSA-C), 6 PD and 5 HC; Validation: 34 MSA, 31 PD, and 30 HC.	TLDA (754 miRNAs) + rt-qPCR (internal control: miR-17 and miR-151-3p)	MSA+PD vs. HC: higher miR-24, miR-223*, and miR-324-3p; lower miR-339-5p. MSA vs. PD: higher **miR-24**, miR-34b, and miR-148b. MSA vs. HC: higher **miR-24**, miR-148b, miR-223*, miR-324-3p; lower miR-339-5p. PD vs. HC: higher **miR-24**, miR-223*, miR-324-3p; lower miR-30c, miR-148b.
Kume (2018), Japan [35]	Serum	10 MSA (5 MSA-P and 5 MSA-C) and 6 HC.	microarray (668 miRNAs)	50 upregulated (miR-16, miR-223 at top) and 17 downregulated miRNAs.
Uwatoko (2019), Japan [36]	Plasma	Microarray: 11 MSA (8 MSA-C and 3 MSA-P) and 6 HC. rt-qPCR: 61 MSA (31 MSA-C and 30 MSA-P), 28 PD, and 28 HC.	microarray (1720 miRNA) + rt-qPCR (internal control: miR-4516)	PD, MSA-P vs. MSA-C, HC: lower miR-671-5p. PD vs. MSA-C, MSA-P, & HC: higher miR-19b-3p. PD vs. MSA-C: higher miR-24-3p.
Pérez-Soriano (2020), Spain [37]	Serum	Microarray: 20 MSA and 40 HC. rt-qPCR: Discovery 20 MSA, 40 HC, and 19 PD; Validation: 20 MSA, 20 HC, and 18 PD.	microarray (2025 pre-miRNA, 2578 mature miRNA) + rt-qPCR (internal controls: miR-320a-3p, miR-6727-5p)	MSA vs. HC: 19 upregulated (miR-16-5p, miR-191-5p, mrR-24-3p, miR-7641, let-7b-5p, miR-425-5p, miR-23a-3p, miR-93-5p, miR-122-5p, miR-103a-3p, miR-4530, miR-17-5p, miR-140-3p, miR-106a-5p, miR-107, miR-25-3p, miR-7704, miR-181a-5p, miR-4487) and 6 downregulated (miR-6797-3p, miR-940, miR-6796-3p, miR-3648, miR-1225-5p, miR-3197) miRNAs. MSA vs. PD (rt-qPCR): miR-191, miR-7641.
Marques (2017), The Netherlands [39]	CSF	17 MSA, 28 PD, and 28 HC.	rt-qPCR (internal controls: miR-16-5p, U6 snRNA)	MSA vs. HC: lower miR-19a-3p, miR-19b-3p, miR-24-3p, miR-34c-5p. PD vs. HC: higher miR-205, lower miR-24-3p.
Starhof (2019), Denmark [40]	CSF, plasma	Discovery: 10 MSA, 10 PSP, 10 PD, and 10 HC. Validation: 29 MSA, 37 PD, 32 PSP, and 23 HC.	microarray (372 miRNAs) + rt-qPCR panel (46 miRNAs; spike-in controls: cel-miR-54-3p, cel-miR-238-3p)	MSA vs. PD: (CSF) lower miR-9-3p, higher miR-106b-5p; (plasma) miR-92-3p, miR-10a-5p, miR-1-3p. MSA vs. HC: (CSF) higher miR-7-5p and miR-34c-3p, lower let-7b-5p; (plasma) miR-19b-3p, miR-34c-3p, miR-99a-5p. PD vs. HC: (CSF) higher miR-7-5p and miR-331-5p, lower miR-145-5p; (plasma) miR-19b-3p, miR-34c-3p. PD vs. PSP (CSF) higher miR-106-5p; (plasma) miR-219-5p.
Ubhi (2014), USA [47]	Human brain (frontal cortex)	3 MSA, 3 AD, 3 DLB, 3 CBD, 3 PSP, and 4 HC.	PCR array for MSA human and mice brain (88 miRNAs); microarray for all human brain (543 miRNAs); rt-qPCR (control: N.A.)	MSA vs. HC: (rt-qPCR) higher miR-96; (microarray and PCR array) upregualted 47 miRNAs.
Lee (2015), USA [49]	Human brain (cerebellum)	4 MSA and 4 HC.	microarray (866 miRNAs) + rt-qPCR (internal control: sno202 RNA)	MSA vs. HC (microarray): 2 upregulated miRNAs (miR-199a-5p, miR-202); 9 downregulated miRNAs (miR-129-3p, miR-129-5p, miR-132, miR-206, miR-337-3p, miR-380, miR-410, and miR-409-5p, miR-433). MSA vs. HC (rt-qPCR): higher miR-202.
Wakabayashi (2016), Japan [50]	Human brain (pons, cerebellum)	11 MSA (5 MSA-C, 6 MSA-P) and 5 HC.	LNA array	MSA vs. HC: (pons) 5 upregulated (miR-1290, miR-21-5p, miR30b-5p, miR-4428, miR-23a-3p) and 33 downregulated (miR-128-3p, miR-371b-3p, miR-3928-3p, miR-1915-3p, miR-129-2-3p, miR-1203, miR-584-5p, miR-1910-5p, miR-675-5p, miR-149-5p, miR1233-3p, miR-3173-5p, miR-1539, miR-513a5p, miR-3663-5p, miR4723-3p, miR-4739, miR-4440, miR-1909-5p, miR-129-5p, miR330-5p, miR-572, miR4632-3p, miR-940,miR-1231, miR-124-3p, miR-34a-5p, miR-210-3p, miR-4687-5p, miR127-3p, miR-138-5p, miR-379-5p, and miR219a-5p) miRNAs; (cerebellum) 5 upregulated ((miR-4428, miR4732-5p, miR-1290, miR-3619-3p, miR4725-3p) and 18 downregulated (miR-4739, miR-4726-3p, miR1228-3p, miR-346, miR-134-5p, miR-1233-3p, miR-484, miR-138-5p, miR-132-3p, miR3663-5p, miR-4440, miR-3184-5p, miR-557, miR-3907,miR-129-5p, miR-219a-2-3p, miR129-1-3p, miR-129- 2-3p) miRNAs.
Valera (2017), USA [51]	Brain (striatum)	17 MSA (MSA-P) and 7 HC.	rt-qPCR (internal control: U6 snRNA)	MSA vs. HC (striatum): higher miR-let-7b, miR-101; lower miR-34c.

Abbreviations: CBD, corticobasal degeneration. DLB, dementia with Lewy bodies. MSA, multiple system atrophy. PSP, progressive supranuclear palsy. TLDA, TaqMan Low-Density Array.

Valera et al. and colleagues focused on a limited list of known autophagy-related miRNAs, including miR-let-7b, miR-30a, miR-34c, miR-96, miR-101, and miR-183 in striatum from 17 cases of MSA (all MSA-P) and 7 cases of HC [51]. They found significantly increased levels of miR-let-7b and miR-101, and decreased levels of miR-34c in MSA. Comparatively, miR-let-7b was also upregulated in serum, and a downregulated miR-34c-5p, but upregulated miR-34c-3p was identified in the CSF from patients with MSA in other miRNA studies [37,39,40]. The authors focus on miR-101 due to its significant association with autophagic impairment in human cancer models and tested putative targeted genes such as RAB5A, MTOR, ATG4D, and STMN1 according to the confidence obtained from the support vector regression scores [52]. Further functional studies demonstrated that miR-101 overexpression in the oligodendroglial cell line inhibited autophagy and worsened the accumulation of *α*-syn. Moreover, the anti-miR-101 injection in MSA transgenic mice showed attenuation of autophagic deficits and *α*-syn aggregation. The framework of this study demonstrates an optimal, sophisticated translational study to explore the possible therapeutic role of differentially expressed miRNAs from clinical samples. Moreover, Kim et al. conducted an advanced mRNA-miRNA network analysis using the same cohort utilized by Valera et al., including 11 patients with MSA (all MSA-P) and 7 patients with HC [51,53]. The original target, miR-101, was not emphasized. In contrast, a list of neuroinflammation-related miRNAs, including miR-124–3p, miR-19a-3p, miR-27b-3p, and miR-29c-3p, was established, identifying essential miRNAs altered in the MSA striatum. The upregulation of miR-let-7b, miR-17 family, miR-19a-3p, miR-24, and miR-181 family (miR-181a-5p, b-5p, c-5p) and downregulation of miR-29c-3p, and miR-124-3p were also addressed, echoing previous findings [51]. Interestingly, the miR-181 family has key roles in regulating various key biochemical processes, such as mitochondrial dysfunction (miR-181a/b), autophagy deficits (miR-181a), and immune response (miR-181c) [54,55,56,57]. This multiverse role of one miRNA family indicates the difficulty of identifying single-disease-specific miRNA regulators, but also indicates the potential for one-on-multiple therapeutic interactions of miRNA therapy in the future.

In summary, these studies focused on brain tissue indicate the possibility of using multi-disease and cross-species approaches to examine versatile temporal changes within variable brain regions and conduct miRNA research in neurodegenerative diseases. However, they also highlight the difficulty of miRNA studies due to a lack of consensus across studies in miRNA identification, selection, quantification, and validation. Nonetheless, the performance of a precise and reliable differential diagnosis biomarker requires multi-cohort, longitudinal follow-up studies to yield replicable results [58]. The power of discrimination stems from the use of receiver-operating characteristic curves to reduce false-positive rates in low-prevalence MSA; however, this process is rarely mentioned in the articles reviewed here.

### 3.3. Alterations to MicroRNA in Cellular Models of MSA

Although current cellular models of MSA still require further refinement to fully recapitulate the pathophysiological alterations observed in MSA brains, non-neuronal or neuronal cells other than oligodendroglial lineage cells have nonetheless provided valuable insights into the roles of brain-derived miRNAs in disease pathogenesis. The identification of differentially expressed brain-derived miRNAs, together with functional studies in cellular models of oligodendrocyte myelination, greatly advances our knowledge of disease-relevant miRNAs and underscores their potential as therapeutic targets for MSA. Generally, two main approaches have been explored: miRNA replacement therapy for down-regulated miRNAs and anti-miRNA therapy for upregulated miRNAs. miRNA replacement therapy involves the delivery of synthetic miRNA mimics to restore the function of downregulated miRNAs, whereas anti-miRNA therapy uses oligonucleotides complementary to specific miRNAs to inhibit their function [59]. To date, only two studies have performed functional experiments on differentially expressed miRNAs identified from clinical biospecimens.

Ubhi et al. demonstrated the functional role of miR-96 since two putative target genes of miR-96, SLC1A1, and SLC6A6, can be downregulated after the transfection of miR-96, as reported by the Luciferase reporter activity in HEK293 (human embryonic kidney 293) cells [47]. KEGG pathway analysis revealed that the miR-183 cluster regulates genes involved in oligodendrocyte maturation and myelination in sensory neurons and the cornea, suggesting their potential role in MSA pathogenesis. Since the target genes of miR-96, SLC1A1 and SLC6A6 participate in glutamate and taurine transportation in the CNS, these findings shed light on the pathoetiology of MSA. Similarly, Valera et al. demonstrated that the lentiviral delivery of miR-101 antagomir alleviates autophagic deficits and alleviates the *α*-syn pathology in both lentiviral a-syn-transfected oligodendroglial cell lines GC-4 and MBP-a-syn in a transgenic mouse model of MSA [51].

Future studies of miRNAs derived from the brain in MSA would benefit from employing ultra-microscaled, cell-specific, and spatial omics-based techniques to elucidate the temporal dynamics and spatial distribution of MSA-related miRNAs. Expanding our understanding of miRNA delivery techniques and crossing the blood–brain barrier will enable cell-specific navigated vehicles such as lipid nanoparticles or novel viral vectors to pave the way for miRNA-based therapy in MSA and other CNS diseases [60,61,62].

## 4. MicroRNAs in the Pathophysiology of MSA

We previously summarized patient-derived microRNAs that are differentially expressed in MSA compared to HC and other disease control groups (i.e., PD). For miRNAs that are more frequently dysregulated in biofluids of brain-derived MSA biospecimens, their associated pathology-related cellular functions have been reviewed previously [63]. Most existing studies on these differentially expressed miRNAs primarily focus on synucleinopathy in disease models. Here, we highlight three additional pathogenic pathways that are particularly relevant to MSA: autophagy dysfunction, oligodendroglial maturation impairment, and microglia-mediated neuroinflammation.

### 4.1. Autophagy-Associated miRNAs

The autophagose–lysosome pathway is one of the pathoetiology-relevant mechanisms in MSA. In the serum-derived miRNA study led by Kume et al., they discovered that putative genetic targets of 6 out of 50 upregulated miRNAs (miR-17, miR-20a, miR-24, miR-25, miR-30d, and miR-451) are involved in autophagy-related genes [35]. Recent studies also implicate miR-16-5p, or the miR-16 family, in the regulation of α-synuclein clearance through autophagosome-related pathways. This was demonstrated in a non-neuronal cell line, showing that miR-16-5p overexpression promotes autophagy via inhibiting the NF-κB signaling pathway [64]. The level of the autophagosomal protein marker LC3 was also found to increase in GCI-positive oligodendrocytes in the brains of MSA patients [65]. However, only miR-16 was annotated and found to be significantly increased in the plasma of MSA patients using rt-qPCR. This finding provides an insight into how miR-16-5p derived from the miR-16 family may be activated downstream to stimulate the autophagosomal function in response to increased α-synuclein cellular aggregation. Interestingly, autophagy–lysosome pathways can be classified into two categories: chaperone-mediated autophagy and macroautophagy. The specific impact of PFF and TPPP/P25α on those two categories is demonstrated [66].

### 4.2. Oligodendrocyte Dysfunction and Demyelination-Associated miRNAs

Oligodendrocyte (OL) dysfunction is a central feature of MSA pathology, contributing to demyelination and neurodegeneration [67]. Several stage-specific miRNAs have been implicated in the regulation of oligodendrocyte lineage differentiation, survival, and myelination [68]. For example, three OL-enriched microRNAs, miR-138, miR-219, and miR-338, have been identified as critical regulators of oligodendrocyte precursor cell (OPC) differentiation for unmyelinated and myelinated mature oligodendrocytes (OL) [69]. The biological function of miR-219 has been examined in more detail in the OL developmental process. At the transformation stage of OPC to OL, miR-219 promotes this process by targeting several downstream substances, such as Sox6 and Hes5, which are key negative regulators of oligodendrocyte differentiation [70]. In addition, miR-219 has multiple implications in other stages of OPC lineage maturation. For instance, it promotes cellular polarity in neural precursor cells of the Zebrafish model and maintains myelin homeostasis by targeting the fatty acid elongase, EVOLV7, at the matured myelinated OL phase [71,72]. Notably, in miRNA studies on brain tissue by the Japanese group, miR-138 was both downregulated in the cerebellum and pons from MSA versus HC, while miR-219a-3p was only downregulated in the cerebellum but not in the pons [50].

Several aforementioned altered miRNAs are also listed in OL development and differentiation [68]. For example, miR-184 promotes differentiation of neural progenitor cells to OPC lineage cells rather than astrocyte or neuron lineage cells. It was found at lower levels in CSF sampled from MSA compared to HC patients [40,73]. Moreover, some miRNAs may be compensatorily elevated to rescue or attenuate the pathophysiology of MSA. For example, miR-202-3p, which is upregulated in MSA brains (Lee et al., 2015), can mitigate the negative effect of IL-1-related inflammation on OPC proliferation and differentiation [49,74]. Moreover, Cheng et al. showed that miR-124 promotes neuron differentiation and induces OL demyelination; by contrast, the level of miR-124-3p is downregulated in the pons of MSA [50,75]. MiR-23a-3p, upregulated in pons of MSA (Wakabayashi et al., 2016), has been implicated in the regulation of oligodendrocyte survival under stress conditions since miR-23a protects OL from apoptosis [50,76].

A fundamental enigma exists regarding the initial origin of GCI and how precursor cells develop into immature and mature oligodendrocytes at various stages [77]. Joint research involving post-mortem brain tissues and human-derived pluripotent stem cells (hiPSC) is crucial to understand how neurons and Ols are induced and co-cultured with other types of glial cells, mimicking the temporal change in intracerebral microenvironments [78,79,80].

### 4.3. Neuroinflammation-Related miRNAs

Extensive neuroinflammation is a prominent feature of MSA pathology, characterized by microglial activation and astrogliosis in affected brain regions [81]. Several miRNAs have been implicated in the regulation of neuroinflammatory responses in MSA, including miR-124, miR-146a, and miR-155. Ponomarev et al. demonstrated that miR-124 promotes microglial quiescence by targeting the transcription factor C/EBP-α [82]. MiR-146a is a well-established regulator of innate immune responses and has been implicated in the modulation of microglial activation [83]. miR-155 is another key regulator of neuroinflammation, promoting microglial activation and pro-inflammatory cytokine production [84]. Interestingly, all miR-124, miR-146a, and miR-155 expression levels are significantly elevated in the frontal cortex of MSA patients, correlating with increased microglial activation and neuroinflammation in this region [47].

Although most of the aforementioned differentially expressed miRNAs in MSA biofluids and brain tissues lack functional experiments to validate their biological influence in MSA cell or animal models, previous basic studies hint at their specific role in the pathophysiology of MSA. Notably, the negative impact of microglia-related neuroinflammation on oligodendroglial myelination has been noticed recently [85,86]. For example, Ji. et al. found that activated microglia-secreted exosomes are enriched with miR-615-5p, which is directly bound to the 3′-UTR of the myelin regulator factor (MYRF) and interferes with OPC maturation [87]. Further investigation of the single or combined effects of miRNAs on microglia–glia interactions could increase our understanding of this complex phenomenon in MSA.

In addition, more significant changes occur in the MSA transcriptome and epigenome compared to miRNA and other non-coding RNA categories, such as DNA methylation, histone modification, and chromosomal structure change [88]. DNA methylation can also be modulated by certain miRNA expressions and functions. For example, the overexpression of miR-29 can increase CG methylation, dampen neuronal activity, and impair brain maturation in an animal model [89]. Considering the finding that miR-29c-3p is downregulated in the striatum of MSA, miRNA-associated epigenetic modulation in MSA also warrants further emphasis in the future [51].

## 5. Conclusions and Challenges

Dysregulated miRNA profiles have been observed in MSA biofluids and brain tissues, correlating with key pathological features, including α-synuclein aggregation, autophagic deficits, oligodendrocyte dysfunction, and neuroinflammation. The identification of specific miRNA signatures associated with MSA may provide valuable biomarkers for the diagnosis and prognosis of the disease. We summarize differentially expressed miRNAs identified and validated in distinct specimens (i.e., serum, plasma, CSF, and several brain regions).

**Figure 1:**
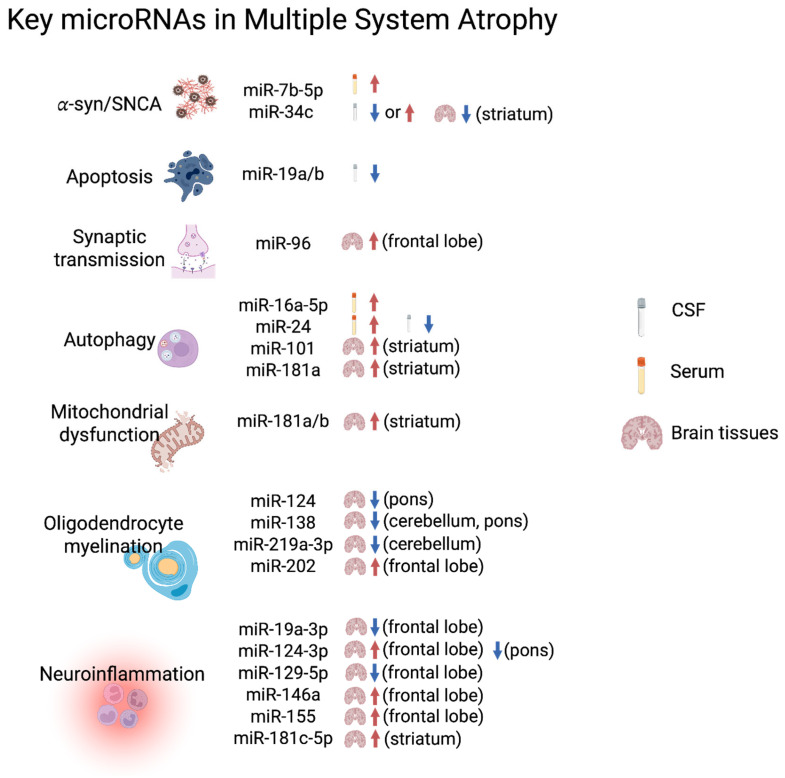
A graphical summary of key microRNAs in MSA. Commonly differentially expressed microRNAs in MSA studies considering biofluids, including serum and CSF, and different regions of brain tissues, such as the frontal lobe, striatum, cerebellum, and pons, are categorized based on their biochemical functions, such as alpha-synuclein production or aggregation, synaptic transmission, autophagic impairment, mitochondrial dysfunction, oligodendrocyte development, demyelination process, and neuroinflammation. Red and blue arrows indicate the upregulation or downregulation, respectively, of microRNAs in each study. Abbreviations: α-syn, alpha-synuclein; CSF, cerebrospinal fluid. (Image was created with BioRender.com).

Some commonly identified miRNAs have been determined; the biological pathways they participate in are partly supported by other experimental evidence (Figure 1). For instance, miR-7 and miR-34c are involved in *SNCA* or *α*-syn overexpression; miR-19a and miR-19b are associated with apoptosis; and miR-96 participates in synaptic transmission. miR-16-5p, miR-101, and miR-181a are related to autophagic impairment; miR-181a/b and miR-202 are found in mitochondrial dysfunction; miR-138, miR-219 are associated with oligodendrocyte proliferation; and miR-19a-3p, miR-124–3p, miR-129-5p, miR-146a, miR-155, miR-181c, miR-27b-3p, and miR-29c-3p occur in excessive or aberrant neuroinflammation responses. Their functional roles are mostly not performed using MSA cell or animal models, except for miR-101, which does not align with potential therapeutic uses [51].

The selection of human study materials further constrains miRNA research in MSA. Post-mortem brain samples predominantly reflect late-stage pathology, whereas CSF sampling offers the potential to assess molecular changes across different phases of disease progression. Nevertheless, most miRNA studies on MSA have not incorporated stratification by disease stage when analyzing either the brain tissue or CSF. This methodological oversight likely contributes to the pronounced inter-study heterogeneity observed in reported miRNA profiles. Consequently, incorporating disease stage and carefully defining disease progression should be prioritized in future investigations evaluating miRNAs as biomarkers or therapeutic targets.

Heterogeneity in detection platforms and control miRNA normalizers are major disadvantages and prevent the development of universal, precise, and reliable miRNA biomarkers to aid in clinical diagnosis and disease monitoring.

Regarding miRNA-based therapy, several challenges must be addressed before clinical translation is possible. The BBB poses a significant obstacle to the delivery of miRNA-based therapeutics to the CNS. Various strategies have been explored to overcome this barrier, including the use of nanoparticles, exosomes, and viral vectors [90]. Currently, no miRNA-related clinical trials have been conducted on MSA as PD [91]. Another challenge is the potential for off-target effects due to the broad regulatory roles of miRNAs. A single miRNA can regulate hundreds of target genes, potentially leading to unintended consequences when modulating miRNA levels. Strategies to enhance the specificity of miRNA-based therapeutics, such as the use of target site blockers, are being explored [59]. Intervention timing is also critical, as the effectiveness of miRNA-based therapies may depend on the disease stage. Early intervention, before extensive neurodegeneration, may offer the greatest therapeutic benefits. However, the lack of reliable biomarkers for early MSA diagnosis remains a significant challenge.

Several issues regarding the translational potential of MSA-specific miRNAs remain unsolved. Developing efficient delivery systems across the BBB for miRNA-based therapeutics could facilitate translational studies [92]. However, challenges such as efficient delivery to the CNS and potential off-target effects must be addressed before clinical usage is possible. Exosomes and extracellular vesicles for miRNAs in MSA and other neurodegenerative diseases are being explored because they can freely cross the BBB. Combination therapies targeting multiple dysregulated miRNAs simultaneously should also be explored. A better understanding of the complex interplay between miRNAs, genetic susceptibility, and environmental factors may refine the framework of MSA pathogenesis and open new avenues for lifestyle modification as a form of treatment and even prevention.

Future research still needs to elucidate substantial major obstacles. Comparing MSA-specific miRNAs in disease-specific OL cell lines or hiPSC-derived cell models, including OL and other neuronal cell types such as Purkinje cells or astrocytes, could address the cell-specific origin of alpha-synuclein aggregation in MSA. Identifying specific miRNA signatures is crucial not only for the diagnosis of disease but also for prognosis. The accurate sensitivity, specificity, and diagnostic power to differentiate MSA from other alpha-synucleinopathies, such as PD and DLB, are essential before commercialization. Advancing and standardizing the detection paradigm of miRNAs could help draw conclusions.

Overall, to enhance the reliability and clinical translational potential of miRNA-based biomarkers for multiple system atrophy, future studies should prioritize methodological standardization, multi-country multi-center longitudinal study designs, and the integration of emerging multi-omics and spatial approaches. Given that most current studies are limited by small sample sizes and restricted to single countries or relatively homogeneous populations, international collaborative efforts adopting uniform diagnostic criteria, such as the Movement Disorder Society Criteria for the Diagnosis of Multiple System Atrophy [93], are essential to increase cohort size, ensure diagnostic accuracy, and improve generalizability. In parallel, the establishment of an open access centralized miRNA database for data sharing and integrative analysis would facilitate cross-study harmonization and accelerate the identification of robust and clinically relevant miRNA biomarkers.

This review exclusively summarizes MSA-specific circulating biofluids and brain-derived miRNAs across datasets from a disease-specific perspective rather than as a typical PD-centered review article. Definite and conclusive miRNA-related biomarkers of MSA are currently lacking. The updated concepts of different miRNA detection platforms and statistics remind us of the complexity of data interpretation in the field. Abundant neuroinflammation and demyelination-associated miRNAs in previous studies highlight the heterogeneous glial involvement in MSA pathology. Overall, methodological standardization, multi-center longitudinal studies, and the integration of emerging multi-omics or spatial approaches could reinforce the specificity, reproducibility, and discriminatory power of any disease-specific miRNAs in future research.

## Data Availability

No new data were created or analyzed in this study. Data sharing is not applicable to this article.
